# Differential utilization of vitamin B_12_-dependent and independent pathways for propionate metabolism across human cells

**DOI:** 10.1016/j.jbc.2024.107662

**Published:** 2024-08-14

**Authors:** Harsha Gouda, Yuanyuan Ji, Sneha Rath, David Watkins, David Rosenblatt, Vamsi Mootha, Jace W. Jones, Ruma Banerjee

**Affiliations:** 1 Departments of Biological Chemistry, University of Michigan, Ann Arbor, Michigan, USA; 2 Department of Pharmaceutical Sciences, University of Maryland School of Pharmacy, Baltimore, Maryland, USA; 3 Howard Hughes Medical Institute and Department of Molecular Biology, Massachusetts General Hospital, Boston, Massachusetts, USA; 4 Broad Institute, Cambridge, Massachusetts, USA; 5 Department of Human Genetics, McGill University, Montreal, Quebec, Canada

**Keywords:** propionate, cobalamin, vitamin B_12_, beta-oxidation, itaconate, odd-chain fatty acids, glycerophospholipids

## Abstract

Propionic acid links the oxidation of branched-chain amino acids and odd-chain fatty acids to the TCA cycle. Gut microbes ferment complex fiber remnants, generating high concentrations of short chain fatty acids, acetate, propionate and butyrate, which are shared with the host as fuel sources. Analysis of vitamin B_12_-dependent propionate utilization in skin biopsy samples has been used to characterize and diagnose underlying inborn errors of cobalamin (or B_12_) metabolism. In these cells, the B_12_-dependent enzyme, methylmalonyl-CoA mutase (MMUT), plays a central role in funneling propionate to the TCA cycle intermediate, succinate. Our understanding of the fate of propionate in other cell types, specifically, the involvement of the β-oxidation-like and methylcitrate pathways, is limited. In this study, we have used [^14^C]-propionate tracing in combination with genetic ablation or inhibition of MMUT, to reveal the differential utilization of the B_12_-dependent and independent pathways for propionate metabolism in fibroblast *versus* colon cell lines. We demonstrate that itaconate can be used as a tool to investigate MMUT-dependent propionate metabolism in cultured cell lines. While MMUT gates the entry of propionate carbons into the TCA cycle in fibroblasts, colon-derived cell lines exhibit a quantitatively significant or exclusive reliance on the β-oxidation-like pathway. Lipidomics and metabolomics analyses reveal that propionate elicits pleiotropic changes, including an increase in odd-chain glycerophospholipids, and perturbations in the purine nucleotide cycle and arginine/nitric oxide metabolism. The metabolic rationale and the regulatory mechanisms underlying the differential reliance on propionate utilization pathways at a cellular, and possibly tissue level, warrant further elucidation.

Propionic acid is a key metabolite in intermediary metabolism and can also derive from the end products of gut metabolism. In the biosphere, propionic acid is an important intermediate in anoxic biomass degradation and a source of biomethane produced by syntrophic propionate-oxidizing bacteria and hydrogenotrophic methanogens (1, 2). In the gut, propionate along with acetate and butyrate are the principle short chain fatty acids produced during the microbial breakdown of complex carbohydrates. Propionate, a fermentation product of Propionibacteria, is enriched in dairy products like yogurt and cheese (3). Following intestinal absorption, propionate enters the liver, which is the primary site for its metabolism in mammals (4).

Propionic aciduria and methylmalonic aciduria are the most frequent branched-chain organic acidurias inherited as inborn errors of metabolism (5, 6). Propionyl-CoA is an intermediate in the catabolism of cholesterol in addition to valine, methionine, isoleucine, threonine, and odd-chain fatty acids in the so-called VOMIT pathway (Fig. 1*A*). Disruption of the B_12_-dependent step in the propionate pathway leads to accumulation of methylmalonic acid. Classically, inborn errors of cobalamin (or B_12_) metabolism associated with methylmalonic aciduria have been characterized by tracking [^14^C]-propionate incorporation into protein in cultured skin fibroblasts (7). Dietary insufficiency or impaired absorption of B_12_, which typically decreases with age, are inversely correlated with methylmalonic acid levels. More recently, methylmalonic acid has been described as an oncometabolite and a metastasis driver (8, 9).

**Figure 1:**
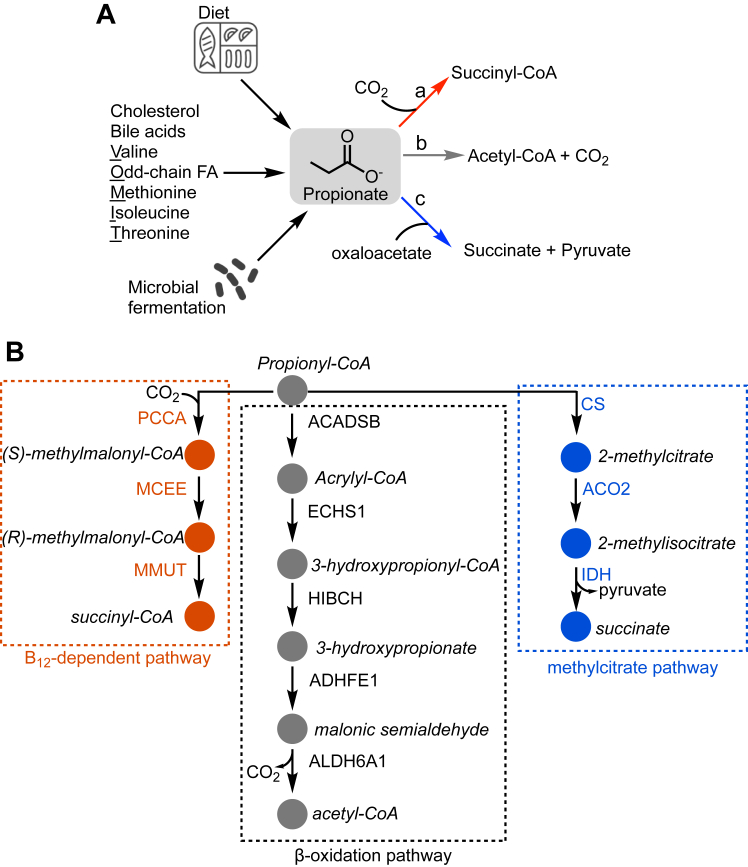
**Sources of propionate-derived propionyl-CoA and routes for its utilization.***A*, propionate is produced by microbial fermentation, cholesterol and bile acid oxidation, catabolism of VOMIT (valine, odd-chain fatty acids, methionine, isoleucine and threonine) metabolites, or obtained from the diet. Propionate can be used as carbon source by one of three major routes: (a) the B_12_-dependent propionate pathway, (b) β-oxidation, and (c) the methylcitrate pathway. *B*, enzymes and intermediates involved in the metabolism of propionyl-CoA *via* the B_12_-dependent, β-oxidation, and methylcitrate pathways. The enzymes in the B_12_-dependent pathway are propionyl-carboxylase (PCC), methylmalonyl-CoA epimerase (MCEE) and methylmalonyl-CoA mutase (MMUT). The enzymes in β-oxidation pathway are: Short and branched chain specific acyl-CoA dehydrogenase (ACADSB), enoyl-CoA hydratase (ECHS1), 3-hydroxyisobutyryl-CoA hydrolase (HIBCH), hydroxyacid-oxoacid transhydrogenase (ADHFE1), malonate semialdehyde dehydrogenase (ALDH6A1). The enzymes in methylcitrate pathway include citrate synthase (CS), aconitate hydratase (ACO2) and isocitrate dehydrogenase (IDH).

The B_12_-dependent pathway for propionate metabolism begins with the carboxylation of propionyl-CoA to (*S*)-methylmalonyl-CoA, catalyzed by propionyl-CoA carboxylase (PCC) (Fig. 1*B*). Isomerization to (*R*)-methylmalonyl-CoA by methylmalonyl-CoA epimerase (MCEE), followed by a 1,2-rearrangement catalyzed by B_12_-dependent methylmalonyl-CoA mutase (MMUT), yields succinyl-CoA. The latter enters mainstream metabolism *via* the Krebs cycle, serving an anaplerotic function (10). Mutations in the genes encoding the α- or β-subunits of PCC leads to propionic acid accumulation while mutations in the genes encoding MMUT and MCEE lead to methylmalonic acid accumulation.

Clinically, propionic aciduria and methylmalonic aciduria are associated with elevated blood levels of 3-hydroxypropionate and 2-methylcitrate (11), which are not intermediates in the canonical B_12_-dependent propionate utilization pathway. 3-Hydroxypropionate is an intermediate in an alternative propionate degradation pathway that is analogous to β-oxidation, which has been described in organisms that do not require B_12_. 2-Methylcitrate can be formed from propionyl-CoA and oxaloacetate in a reaction catalyzed by citrate synthase (CS) (Fig. 1*B*). In the methylcitrate pathway, aconitate hydratase (ACO2) isomerizes 2-methylcitrate to 2-methylisocitrate, followed by the oxidative elimination of pyruvate and succinate, catalyzed by isocitrate dehydrogenase (IDH).

In the β-oxidation-like pathway, propionyl-CoA is converted by short-chain acyl-CoA dehydrogenase (ACADSB) to a reactive acrylyl-CoA intermediate, which undergoes hydration to form 3-hydroxypropionyl-CoA, catalyzed by enoyl-CoA hydratase (ECHS1) (Fig. 1*B*). Subsequent hydrolysis to 3-hydroxypropionate catalyzed by 3-hydroxyisobutryl-CoA hydrolase (HIBCH), and oxidation to malonate semialdehyde, catalyzed by hydroxyacid-oxoacid transhydrogenase (ALDH6A1), yields acetyl-CoA.

In the nematode *Caenorhabditis elegans,* the B_12_-dependent and β-oxidation pathways for propionate catabolism reportedly coexist (12). The β-oxidation genes are transcriptionally activated in *C. elegans* under B_12_-deficient conditions, while the B_12_-dependent pathway is favored when the cofactor is available (Fig. 1*A*, *paths a,b*). In the arthropod, *Periplaneta americana*, propionate is converted to acetyl-CoA, eliminating CO_2_ *via the* β-oxidation-like pathway (Fig. 1*A*, *path b*) (13). In contrast, in *Saccharomyces cerevisiae*, propionate is metabolized *via* the methylcitrate pathway, forming pyruvate and succinate (Fig. 1*A*, *path c*) (14).

Exposure of mice to the environmental toxin, 2,3,7,8-tetrachlorodibenzo-*p*-dioxin, decreases serum B_12_ levels and inhibits MMUT function (15). These observations were explained by the increased synthesis of itaconate, a suicide inhibitor of MMUT (16). Elevated levels of *S*-(2-carboxyethyl)-L-cysteine observed in TCDD-exposed mice, reportedly resulted from the reaction of cysteine with acryly-CoA. Elevation of 3-hydroxypropionate levels in propionic aciduria and methylmalonic aciduria patients (17, 18), and detection of the acrylyl-CoA conjugation product of cysteine in 2,3,7,8-tetrachlorodibenzo-*p*-dioxin-exposed mice, suggests that propionate metabolism is rerouted through the β-oxidation-like pathway under these conditions. Additionally, elevated methylcitrate levels in patients with propionic aciduria and methylmalonic aciduria indicates involvement of the methylcitrate pathway at least under pathological conditions (17, 18).

The quantitative significance of short-chain fatty acids produced by gut microbes to host metabolism is poorly understood. Propionate concentration is estimated to be 4.5 ± 0.4 mM in the murine cecum, *that is*, the compartment where it is primarily produced, and it can be taken up and assimilated into glucose by the host (19). Furthermore, propionyl-CoA can substitute for malonyl-CoA, leading to odd-chain fatty acid synthesis (20). In fact, increased dietary fiber intake increases propionate and plasma pentadecanoic (15:0) and heptadecanoic (17:0) acid (20). The metabolic routes that are used for propionate utilization by different cell types in host, which interface with microbiota, are however, poorly understood.

In this study, we report the differential utilization of propionate metabolic pathways in cell lines derived from colon epithelium *versus* skin fibroblasts. We find that human fibroblast HFF1 cells metabolize propionyl-CoA predominantly *via* the B_12_-dependent pathway, consistent with the clinical use of patient-derived skin cells to evaluate cobalamin disorders (7). In contrast, the colorectal adenocarcinoma cell line, HT-29, uses the B_12_ route sparingly, if at all. We demonstrate the utility of itaconate, a mechanism-based inhibitor of MMUT (16, 21), to evaluate the B_12_-dependent contribution to propionate metabolism, which confirms its variable utilization across cell lines. In different cell lines, propionate induces changes in mRNA and protein expression that are correlated with the use of the β-oxidation-like (HT-29) *versus* B_12_-dependent (HFF1) pathways. Metabolomics and lipidomics analyses reveal increases in odd-chain glycerophospholipids in response to propionate, and changes that map to the purine nucleotide cycle and to arginine and nitric oxide metabolism.

## Results

### Variability in B_12_-dependent propionate metabolism across human cell lines

**Figure 2:**
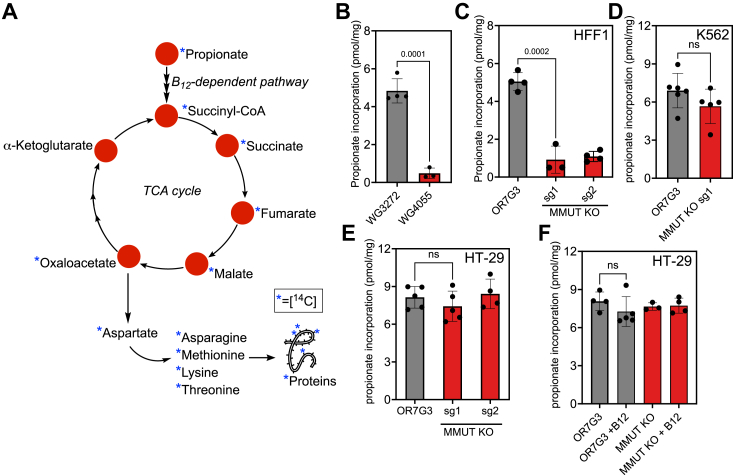
**Differential MMUT dependent propionate metabolism in human cell lines.***A*, scheme tracing [^14^C]-propionate incorporation into proteins *via* intermediates in the TCA cycle. Additionally, pyruvate eliminated from 2-methylisocitrate can be converted to alanine and contribute to protein labeling but is not shown for the sake of figure clarity. *B*, propionate incorporation into proteins in control (WG3272) and fibroblasts from a MMUT deficiency patient (WG4055). The data represent the mean ± SD of three or four biological replicates. *C*, propionate incorporation in HFF1 cells with MMUT knockout (sg1 and sg2) and a CRISPR control (OR7G3). Data represent the mean ± SD of three or four biological replicates. *D* and *E*, propionate incorporation into proteins in K562 (*D*) and HT-29 cells (*E*) MMUT KOs *versus* the OR7G3 CRISPR controls in the respective cell lines. Data represent the mean ± SD of three or six biological replicates as indicated. *F*, aquocobalamin (1 mg/ml) supplementation did not influence propionate incorporation into proteins in control (OR7G3) or MMUT KO HT-29 cells.

The [^14^C]-propionate incorporation assay in heterokaryons of human skin fibroblasts from patients with methylmalonic aciduria has been used to differentiate between genetic complementation groups in inherited cobalamin deficiency disorders (22). In this assay, radiolabel transfer from [^14^C]-propionate to protein occurs *via* TCA cycle intermediates (Fig. 2*A*). We confirmed that compared to a control human fibroblast cell line (WG3272), propionate incorporation was severely attenuated (10-fold) in a patient line harboring mutations in MMUT (WG4055; c.572C > A, c.682C > T) (Fig. 2*B*). The data confirm that the B_12_-dependent route accounts for the majority of propionate metabolism in primary skin fibroblasts.

Next, the prevalence of B_12_-dependent propionate metabolism was evaluated across other human cell lines. MMUT deficiency was introduced *via* CRISPR knockout (KO) in foreskin fibroblast (HFF1), colorectal adenocarcinoma (HT-29), and lymphoblast (K562) cell lines. Targeted KO of MMUT with two guide sequences (sg1 and sg2) was confirmed by Western blot analysis (Fig. S1). Compared to the CRISPR OR7G3 control (negative control with the guide sequence for olfactory receptor family seven subfamily G3), MMUT KO in HFF1 cells exhibited an approximately 6-fold lower propionate incorporation into proteins (Fig. 2*C*). Surprisingly, the MMUT KO had no effect on propionate incorporation in K562 or HT-29 cells (Fig. 2, *D* and *E*), indicating that alternate pathways for propionate utilization were operative. To exclude the possibility that B_12_ is limiting, HT-29 cells were cultured in medium supplemented with aquocobalamin (1 mg/ml), which however, had no effect (Fig. 2*F*).

### Itaconate inhibits MMUT-dependent propionate metabolism

**Figure 3:**
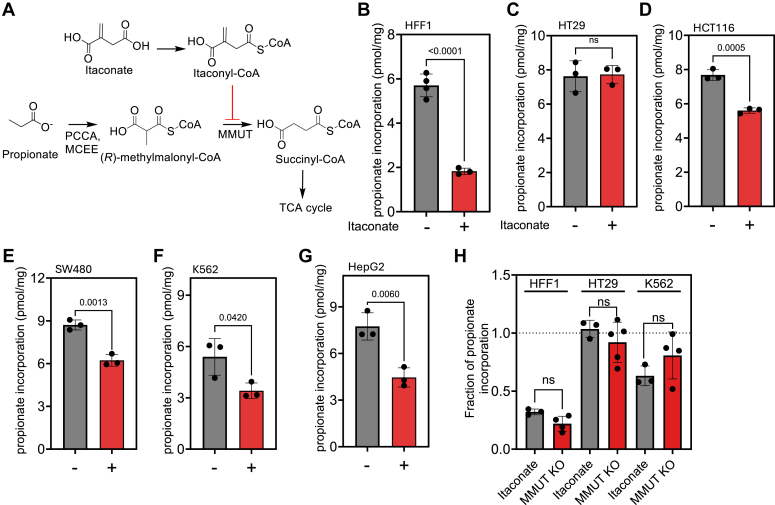
**Itaconate inhibits MMUT-dependent propionate metabolism.***A*, Itaconyl-CoA derived from itaconate is postulated to prevent the entry of propionate carbon into the TCA cycle by inhibition of MMUT. PCC, MCEE, and MMUT denote propionyl-carboxylase, methylmalonyl-CoA epimerase, and methylmalonyl-CoA mutase respectively. *B*, the effect of 1 mM itaconate for 24 h differentially affects propionate incorporation into proteins in HFF1 (*B*), HT-29 (*C*), HCT116 (*D*), SW480 (*E*), K562 (*F*), and HepG2 (*G*) cells (n = 3 independent experiments, mean ± SD). *H*, comparison of fractional inhibition of propionate incorporation by itaconate (1 mM) *versus* MMUT KO (sg1) in HFF1, HT-29 and K562 cells. The dashed line (at y = 1.0) corresponds to no inhibition, indicating that the B_12_ pathway does not make a measurable contribution.

We evaluated the efficacy of pharmacological inhibition of MMUT in the propionate incorporation assay as an alternative to genetic ablation (Fig. 3*A*). The thioester derivative of itaconate, an immunometabolite, is a suicide inhibitor of MMUT (16, 21). Itaconate (1 mM) treatment dramatically decreased propionate incorporation in HFF1 but not in HT-29 cells (Fig. 3, *B* and *C*), qualitatively phenocopying the CRISPR KO data (Fig. 2, *C* and *E*). Using this approach, we evaluated the significance of B_12_-dependent propionate metabolism in two other colorectal carcinoma cell lines, HCT116 and SW480, in addition to K562 and HepG2 cells (Fig. 3, *D*–*G*). The degree of inhibition of propionate incorporation between the itaconate treatment *versus* MMUT KO was not significantly different in HFF1, K562 or HT-29 cells (Fig. 3*H*), establishing itaconate as a pharmacological probe for evaluating B_12_-dependent propionate metabolism.

### Differential propionate-induced responses in gene expression

**Figure 4:**
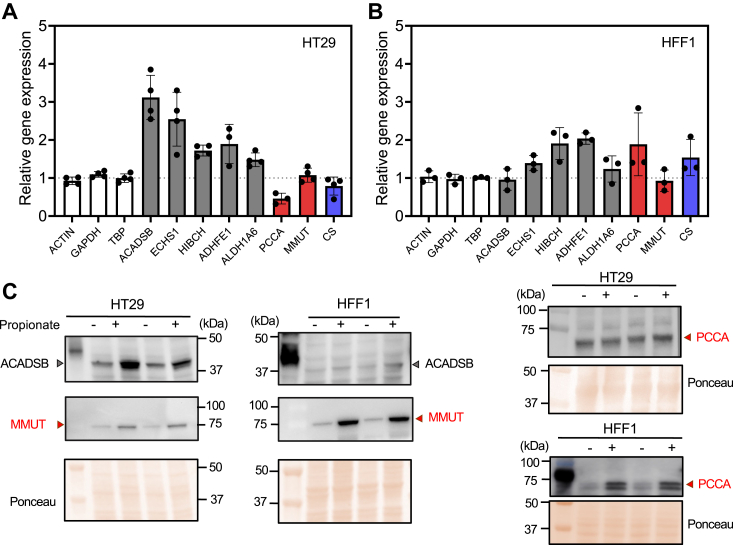
**Propionate preferentially induces genes in the β-oxidation-like pathway in HT29 cells.***A and B*, RT qPCR analysis of mRNA levels for genes involved in propionate metabolism in HT-29 (*A*) and HFF1 (*B*) cells treated with 5 mM propionate for 24 h. The data represent the mean ± SD of three or four biological replicates. Actin, glyceraldehyde 3-phosphate dehydrogenase (GAPDH) and TATA-binding protein (TBP) were included as controls. *C*, Western blot analysis of ACADSB, PCCA and MMUT in HT-29 and HFF1 cells treated as described in *A* and *B*.

To further investigate differences between the HT-29 and HFF1 cell lines, we used real-time quantitative PCR and Western blot analysis to examine transcriptional and translational regulation of key components in each of the three routes for propionate utilization. Increased mRNA levels for genes involved in the β-oxidation pathway (ACADSB, ECHS1, HIBCH, and ADHFE1) were seen in HT-29 cells in response to propionate (Fig. 4*A*). Within this group, mRNA encoding ACADSB, which commits propionate to β-oxidation, showed the largest (3-fold) increase. In contrast, the transcript encoding the α-subunit of propionyl-CoA carboxylase (PCCA), which catalyzes the committing step in B_12_-dependent pathway, was decreased 2-fold in response to propionate. In contrast, mRNA levels for citrate synthase, which commits propionate to oxidation *via* the TCA cycle were unchanged.

In HFF1 cells, mRNA levels for ACADSB were unchanged while those encoding two downstream enzymes in the β-oxidation-like pathway, HIBCH and ADHFE1 were each elevated 2-fold, in response to propionate (Fig. 4*B*). PCCA mRNA was 2-fold higher but citrate synthase was not significantly different in propionate-treated *versus* untreated cells (Fig. 4*B*).

At a protein level, propionate induced a significant increase in ACADSB but not PCCA in HT-29 cells (Figs. 4*C* and S2). A faint ∼80 kDa, which increased in intensity in response to propionate, indicated very low expression of MMUT in HT-29 cells. In HFF1 cells, ACADSB levels were very low and unchanged, while PCCA and MMUT levels increased in response to propionate.

### Propionate-induced changes in the metabolome

**Figure 5:**
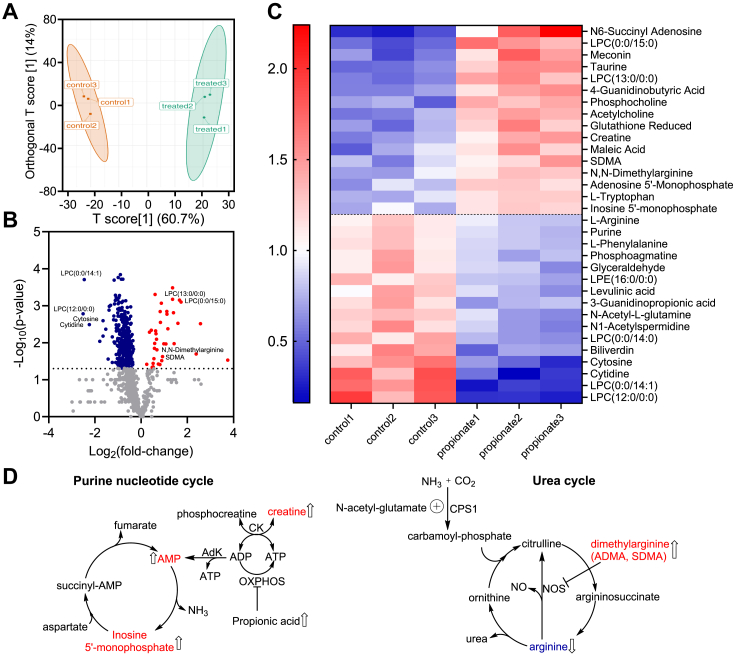
**Propionate induces widespread metabolic changes.***A*, orthogonal partial least square-discriminant analysis (OPLS-DA) of metabolomics data from HT-29 cells treated with propionate (5 mM, 24 h) compared to untreated cells. *B*–*C*, differential metabolite analysis indicates significant differences (*p* < 0.05) in 37 upregulated and 413 downregulated metabolites displayed in volcano plot (*B*) and heatmap (*C*) format in the propionate *versus* untreated samples described in A. *D*, scheme showing increased levels of purine nucleotide intermediates, and the connection to creatine metabolism *via* creatine kinase (CK) and adenylate kinase (AdK), which can regenerate ATP from ADP when oxidative phosphorylation is inhibited. In the urea cycle, nitric oxide synthase (NOS) inhibitors,asymmetric (ADMA) and symmetric (SDMA) dimethylarginine levels are elevated, while arginine levels are decreased.

We used untargeted metabolomics to examine changes induced by propionate exposure (5 mM, 24 h) in HT-29 cells (Table S1). Orthogonal partial least square-discriminant analysis revealed a clear separation between the untreated and propionate-treated groups (Fig. 5*A*). Phosphocholine, an intermediate in the synthesis of phosphatidylcholine, which is remodeled to lysophosphatidylcholine *via* the action of phospholipase A2, was significantly upregulated in propionate-treated cells. Interestingly, two lysophosphatidylcholines with odd-chain fatty acids (LPC15:0 and LPC13:0) esterified to glycerophosphocholine were enriched while the even-chain derivatives (LPC14:1 and LPC12:0) were decreased in propionate treated cells (Fig. 5, *B* and *C*). Table 1 lists the propionate-induced changes in phospholipid distribution. Other significant metabolite changes mapped to the purine nucleotide cycle (adenylosuccinate, AMP and IMP) or were connected to arginine and nitric oxide metabolism (arginine, N,N-dimethylarginine, symmetric dimethylarginine (SDMA), and 4-guanidinobutanoic acid) (Fig. 5, *C* and *D*).

**Table 1:** Propionate influences phospholipid composition in HT-29 cells

Compound	*p*-value	Fold-change	Type
LPC(0:0/15:0)	7.01E-04	3.16	up
LPC(13:0/0:0)	6.72E-04	2.56	up
LPE(P-17:0)	3.83E-02	1.18	up
PC(16:0/2:0)	5.98E-03	0.76	down
LPE(0:0/20:4)	2.45E-02	0.73	down
LPE(16:1/0:0)	3.02E-02	0.72	down
LPE(20:4/0:0)	1.24E-02	0.72	down
LPE(20:5/0:0)	4.93E-03	0.71	down
LPE(18:1/0:0)	9.52E-03	0.70	down
LPE(22:6/0:0)	3.98E-03	0.68	down
LPE(0:0/20:5)	2.31E-03	0.68	down
LPE(22:5/0:0)	8.16E-03	0.66	down
LPE(20:3/0:0)	3.90E-02	0.66	down
LPE(0:0/20:3)	2.61E-03	0.65	down
LPC(16:1/0:0)	4.49E-02	0.65	down
LPE(0:0/22:4)	2.59E-03	0.64	down
LPE(0:0/18:3)	3.99E-02	0.62	down
PC(8:0/8:0)	4.26E-02	0.62	down
LPE(16:0/0:0)	6.51E-03	0.62	down
LPE(18:2/0:0)	1.96E-02	0.60	down
LPE(O-18:2)	9.47E-03	0.59	down
LPC(0:0/22:6)	3.49E-02	0.59	down
LPE(O-18:1/0:0)	7.54E-04	0.58	down
PI(15:0/2:0)	7.82E-03	0.52	down
LPC(0:0/14:0)	4.94E-03	0.52	down
LPC(O-16:0)	1.24E-03	0.50	down
LPC(0:0/14:1)	1.97E-04	0.18	down
LPC(12:0/0:0)	1.65E-03	0.18	down

Levels of lysophosphatidylcholines (LPC) and phosphoatidylethanolamines (LPE) in HT-29 cells treated with 5 mM propionate detected by MS-based widely targeted metabolomics approach.

### Propionate elicits changes in glycerophospholipid composition

**Figure 6:**
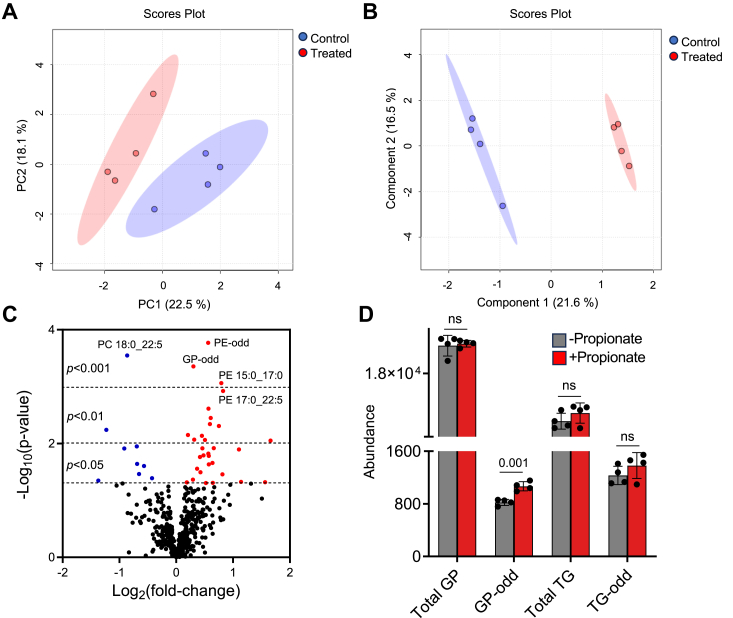
**Propionate alters the lipid composition in HT-29 cells.***A* and *B*, principal component analysis (PCA) and partial least squares discriminant analysis (PLS-DA, Q2 = 0.414) of the lipidomics data indicated that cells treated with and without propionate clustered separately. Data were sum normalized, log transformed and mean centered. The elliptical patterns represent the 95% confidence interval. *C*, Volcano plot display of differentially expressed lipids highlighting fold change and significance. Forty lipids were significantly (*p* < 0.05) up-regulated and nine were down-regulated. PC = glycerophosphatidylcholine, PE = glycerophosphatidylethanolamine, GP = glycerophospholipids. *D*, Lipid abundance for total lipid class population or only considering odd-length acyl chains in untreated (*grey*) *versus* propionate treated (*red*) HT-29 cells. TG = triacylglycerol, ∗∗∗ = *p*-value <0.005, ns = non-significant. The values represent the mean ± SD (n = 4 per group).

We performed high-throughput, targeted lipidomics to investigate changes in lipid composition in HT-29 cells exposed to propionate. In contrast to the metabolomic experiments discussed above, the lipidomic experiments relied on a lipid extraction method that biased lipid detection towards non-polar lipids. This allowed us to generate complementary lipidomic and metabolomic data. The multivariate analysis comparing lipid extracts from control and propionate-treated HT-29 cells revealed distinct lipid compositions between the two groups (Fig. 6, *A* and *B*). This indicated that administration of propionate to cells preferentially impacts the lipid composition in a unique way. The principal component analysis (PCA) plot, as an unsupervised analysis method, revealed the lipid composition of treated and untreated cells was distinct yet not completely separated along the PC1 axis (Fig. 6*A*). This showed that although the lipid compositions are different, the differences were specific and not broad or universal. Next, the data was visualized by a supervised statistical model, partial least squares-discriminate analysis (PLS-DA, Fig. 6*B*) to identify lipid features that potentially distinguish the two groups. The PLS-DA plot had a Q2 score of 0.414, which indicated the model was predictive for using the lipid composition to differentiate between control and propionate-treated cells. The lipid differences between the groups were further visualized using a volcano plot (Fig. 6*C*). A total of 40 lipids were significantly up-regulated and nine were down-regulated (*p* < 0.05) in propionate-treated cells. The most prominent changes were seen in the up-regulation of glycerophospholipids containing odd-length fatty acyl chains (Fig. 6*D*). No other significant changes were observed when considering lipid classes or odd-length fatty acyls from other lipid classes. Table 2 provides the complete list of lipids that were significantly up- or down-regulated in response to propionate.

**Table 2:** Propionate influences odd-length acyl chain gylcerophospholipid composition in HT-29 cells

Lipid ID	*p*-value	Fold change	Type
Hex2Cer d18:1/18:0	2.20E-02	1.56	Up
Hex2Cer d18:1/22:1	1.72E-02	1.34	Up
PC 15:0_15:0	4.92E-03	1.68	Up
PC 15:0_16:0	2.43E-03	1.48	Up
PC 15:0_16:1	8.66E-03	1.42	Up
PC 15:0_18:1	3.55E-03	1.52	Up
PC-odd	8.56E-03	1.25	Up
PE 15:0_16:0	2.33E-02	1.49	Up
PE 15:0_17:0	8.66E-04	1.73	Up
PE 15:0_18:0	4.55E-03	1.51	Up
PE 15:0_18:1	1.65E-02	1.49	Up
PE 15:0_20:1	1.51E-02	1.48	Up
PE 15:0_22:5	7.34E-03	1.37	Up
PE 17:0_18:0	1.22E-02	1.38	Up
PE 17:0_18:1	3.20E-02	1.34	Up
PE 17:0_22:5	1.19E-03	1.77	Up
PE 18:2_20:4	4.73E-02	2.20	Up
PE O-18:0_16:0	7.07E-03	1.15	Up
PE O-18:0_16:1	4.82E-02	1.14	Up
PE O-18:0_18:0	1.62E-02	1.39	Up
PE-odd	1.70E-04	1.48	Up
PI 15:0_20:0	1.27E-02	2.15	Up
GP-odd	4.42E-04	1.23	Up
TG 47:1_16:0	4.32E-02	1.23	Up
TG 48:2_18:1	4.81E-02	2.96	Up
TG 51:2_16:0	3.47E-02	1.76	Up
TG 54:3_18:1	8.92E-03	3.16	Up
TG 54:6_20:5	4.96E-02	1.44	Up
TG 55:1_18:1	4.92E-02	1.56	Up
TG 56:3_18:0	2.77E-02	1.29	Up
TG 60:10_22:6	1.20E-02	1.58	Up
Hex1Cer d18:0/22:0	2.49E-02	0.67	Down
PC 16:1_20:4	1.13E-02	0.62	Down
PC 18:0_18:3	2.28E-02	0.62	Down
PC 18:0_20:5	4.49E-02	0.39	Down
PC 18:0_22:5	2.83E-04	0.55	Down
PC 18:1_22:6	3.45E-02	0.64	Down
PE 16:0_18:3	4.06E-02	0.74	Down
TG 50:2_14:0	1.22E-02	0.53	Down
TG 53:2_17:0	5.75E-03	0.43	Down

Differentially expressed lipids in HT-29 cells treated with 5 mM propionate detected by LC-MS/MS targeted lipidomics.

## Discussion

While toxic at elevated levels, propionate is beneficial to gut health and reportedly has cholesterol-lowering and anti-lipogenic effects (23). Propionate utilization *via* the β-oxidation-like pathway induces mucin synthesis in a goblet cell line *via* hypoxic activation of HIF2α signaling (24). Evaluation of propionate utilization in clinical genetics studies on cobalamin deficiency patients have perforce relied on the B_12_-dependent pathway and revealed its predominance in skin fibroblasts (7). On the other hand, clinical evidence of 3-hydroxypropionate and 2-methylcitrate accumulation in propionic aciduria patients clearly indicates that B_12_-independent pathways are also operational at least under pathological conditions (11, 18). A study of the interorgan exchange of short-chain fatty acids in humans revealed that while propionate produced in the gut is utilized predominantly by liver, there is ∼20% splanchnic release (25). Our understanding of propionate metabolism in peripheral tissues other than fibroblasts is, however, limited.

A GWAS study identified a single nucleotide polymorphism in the gene encoding HIBCH in the β-oxidation-like pathway, which is strongly associated with elevated methylmalonic acid, but is independent of B_12_ status (26). The same set of enzymes operates in the valine and propionate catabolic pathways in which the substrates differ by a single carbon. Reactive intermediates, namely methylacrylyl-CoA and acrylyl-CoA, are formed in the valine and propionate pathways, respectively. Interestingly, mutations in the genes encoding HIBCH and ECHS1 lead to the accumulation of acrylyl-CoA and methylacrylyl-CoA adducts with cysteine and cysteamine, further supporting propionate flux through the β-oxidation-like pathway (27, 28, 29). Using a combination of approaches, our study reveals the quantitative significance of the B_12_-independent pathways for propionate metabolism across multiple cell lines.

Our identification of the flexible utilization of propionate metabolism pathways, suggests a possible rationale for the correlation between elevated levels of methylmalonic acid in blood and risk for tumor development in elderly individuals (9). Decreased secretion of intrinsic factors from gastric parietal cells with age decreases cobalamin absorption (30). We speculate that the loss of MMUT function in the elderly due to low cobalamin might lead to rewiring of propionate metabolism through the β-oxidation-like pathway, which includes a highly reactive acrlyl-CoA intermediate. Increased synthesis of acrlyl-CoA, identified as a carcinogen (31), could play a role in tumor development.

*C. elegans* preferentially utilizes the canonical B_12_-dependent pathway to metabolize propionate and avert the synthesis of toxic acrylate from acrylyl-CoA in the β-oxidation-like pathway (12). However, when B_12_ is limiting, the β-oxidation pathway is transcriptionally upregulated by the nuclear hormonal receptors, NHR-10 and NHR-68 (32). In our study, propionate induced expression of genes encoding β-oxidation-like pathway proteins (Fig. 4). However, humans lack homologs of the NHR-10 and NHR-68 receptors found in *C. elegans* and the underlying mechanism for regulating the β-oxidation-like pathway in response to propionate is not known.

Propionate accumulation in propionic aciduria patients is associated with metabolic hyperammonemia (33). Glutamine is the primary source of ammonia in chronic metabolic acidosis and is cleaved by the successive action of glutaminase and glutamate dehydrogenase (34). Our metabolomics analysis of HT-29 cells exposed to propionate revealed decreased arginine, which has been seen in the plasma of propionic aciduria patients (35). Cytidine (3-fold) and cytosine (5-fold) levels were also significantly lower, suggesting decreased availability of carbamoyl phosphate needed for pyrimidine biosynthesis and ureagenesis (36). The nitric oxide synthase inhibitors N, N-dimethylarginine and symmetric dimethylarginine were elevated, suggesting decreased nitric oxide synthesis (37).

Microbial fatty-acid synthase can reportedly use propionyl-CoA as a primer for the synthesis of odd-chain fatty acids (38). Red blood cell membranes have elevated levels of pentadecanoic and heptadecanoic acid in patients with propionate disorders (39). Our metabolomics and lipidomics data revealed that propionate-treated cells are enriched in glycerophospholipids containing odd-length fatty acids. Of note, the odd-length fatty acid enrichment was exclusively observed in glycerophospholipids and not in other lipid classes, such as glycerolipids (*e.g.*, triacylglycerides). This suggests that incorporation of the odd-length acyl chains is preferentially directed towards membrane and/or structural lipids (*i.e.*, phosphatidylcholine and phosphatidylethanolamine) as opposed to energy storage and metabolism lipids (*i.e.*, triacylglycerides).

Our study reveals that the B_12_ and β-oxidation-like pathways contribute differentially to propionate clearance in different cell types. A notable limitation of our study was that it focused on primary patient fibroblasts and transformed or malignant human cell lines. Investigation of preferred propionate utilization routes at the tissue level will be an important next step for elucidating organ-specific differences. Understanding the mechanism by which propionate induces transcriptional upregulation of the β-oxidation-like pathway in some cell types but not in others, holds therapeutic promise for preventing the toxic buildup of propionate in classical inborn errors of propionate metabolism by activating an alternate route.

## Experimental procedures

### Materials

LC-MS grade acetonitrile (ACN), methanol (MeOH), water (H_2_O), and *n*-propanol were purchased from Fisher Scientific (Pittsburg, PA). HPLC grade tert-Butyl methyl ether (MTBE), chloroform, ammonium format, and formic acid were purchased from Sigma Aldrich (St Louis, MO). EquiSPLASH lipidomix was purchased from Avanti Polar Lipids, Inc. An additional five fatty acid internal standards including docosahexaenoic acid-d_5_, eicosapentaenoic acid-d_5_, arachidonic acid-d_11_, palmitic acid-d_9_ and *α*-linolenic acid-d_5_ were purchased from Cayman Chemical (Ann Arbor, MI). An additional four ceramides including C13 galactosyl(*β*) Ceramide-d_7_ (d18:1-d_7_/13:0), C15 glucosyl(*β*) Ceramide-d_7_ (d18:1-d_7_/15:0), C15 lactosyl(*β*) Ceramide-d_7_ (d18:1-d_7_/15:0) and C13-dihydroceramide-d_7_(d18:0-d_7_/13:0) were purchased from Cayman Chemical (Ann Arbor, MI).

### Cell culture conditions

HFF1, K562, SW480, HCT116, and HepG2 cells were from ATCC and maintained in the DMEM (Gibco, 11995-065) medium. The HT29 cells were maintained in RPMI1640 (Gibco, 11875-093) medium. The control (WG3272) and MMUT deficiency (WG4055) primary fibroblast cell lines were obtained from the Vitamin B12 Clinical Research Laboratory (McGill University) and were maintained in DMEM/F-12 Glutamax (Gibco, 10565-018) medium. All growth media were supplemented with 10% fetal bovine serum (FBS, Gibco, #A5256701), 100 units/ml penicillin, and 100 μg/ml streptomycin. Cells lines were maintained at 37 °C in a humidified atmospheric chamber at ambient O_2_ and 5% CO_2_.

### Generation of MMUT CRISPR-KO cell lines

MMUT knockout in HFF1 and HT29 cell lines were generated using CRIPSR-cas9 genome editing with the following two guide sequences: sg1 5ʹ-ATCATGTAAGAACCTCCCCA-3ʹ, and sg2 5ʹ- GGAGTGAAGCCATTCACACG-3ʹ were cloned into a lentiCRISPRv2 vector (Addgene # 52961). Following purification, the plasmids harboring the MMUT or control (OR7G3: 5ʹ-GGTGAAACAGATGTCGACCA-3ʹ) oligoucleotides were submitted to the Vector Core (University of Michigan) for lentiviral packing. Cells (1 × 10^6^) were seeded in a 6-well plate containing 2 ml of growth medium and transduced with optimized lentivirus titer and selected using 1 μg/ml (HT29) or 0.5 μg/ml (HFF1) puromycin as described previously for *Lb*NOX expression (40). The MMUT CRISPR-KO was validated by Western blot analysis using a commercially available anti-human MMUT antibody (Abcam, ab134956, lot #GR3251447-2).

### Propionate incorporation into proteins

Cells were grown to ∼50% confluency in 6 cm plates. Then, the medium was aspirated, the cells were rinsed with PBS and fresh 2 ml medium containing Earls balanced salt solution (EBSS, Sigma, E6267-500 Ml) supplemented with 5% FBS, 1 μCi/ml [1-^14^C]-propionic acid (American Radiolabeled Chemicals Inc, #ARC0203 A), 0.2 mM unlabeled propionate (Sigma, 402907), 100 units/ml penicillin and 100 μg/ml of streptomycin was added. In the itaconate-inhibition experiments, 1 mM itaconate (Sigma, I29204) was added to the medium. In cobalamin-supplemented conditions, 1 μg/ml aquocobalamin (Sigma-Aldrich, H7126-1G) was added to the medium. Cells were incubated in a humidified chamber for 24 h.

On the next day, the medium was aspirated and cells were rinsed twice with PBS and treated with 500 μl 0.05% trypsin-EDTA (w/v) for ∼10 min at 37 °C. When the cells were detached, 1 ml 15% FBS in PBS was added and the cells were transferred to 2 ml Eppendorf tubes. The tubes were centrifuged at 200 × *g* for 10 min, and the supernatant was discarded. The cell pellet was washed with 1 ml PBS, centrifuged and the supernatant was again discarded. The cell pellet was resuspended in 500 μl 10% (w/v) trichloroacetic acid (Fischer, A323-500), vortexed, and incubated on ice for 10 min. The samples were centrifuged at 200*g* and the supernatant was removed as completely as possibly, leaving the protein precipitate, which was resuspended in 150 μl 1 M NaOH. For each sample, a 100 μl aliquot was added to a vial containing 5 ml scintillation cocktail. The remainder of the sample was used to determine protein concentration using the Bradford reagent (BioRad, Cat #5000006). The radioactive counts per minute (cpm) for samples were determined using the LS6500 scintillation counter (Beckman Coulter). Negative (100 μl 1 M NaOH) and positive (100 μl of media with 1 μCi/ml [1-^14^C]-propionate) controls were included in the scintillation counter.

The radioactive counts in the [1-^14^C]-propionate medium (positive) control were divided by the total propionate concentration ([1-^14^C]-propionate (0.017 mM) + cold propionate (0.2 mM)) to calculate the specific activity (*i.e.*, picomoles of propionate per cpm). The specific activity of the stock [1-^14^C]-propionate (58 mCi/mmol) was then used to calculate the concentration (0.0172 mM) of [1-^14^C]-propionate (1 μCi/ml) in each sample. The total cpm for each sample was multiplied by the number of picomoles of propionate per cpm, to determine the total picomoles of propionate in the protein pellet. For normalization, the total picomoles of incorporated propionate was divided by the amount of protein in the sample, to determine the picomoles of propionate incorporated/mg of protein.

### RNA isolation and qPCR analysis

Cells (6 × 10^ˆ6^) were cultured in 10-cm plates with 10 ml culture medium to a confluency of ∼50%. For propionate treatment, culture media was exchanged with 10 ml EBSS media containing 5% FBS and 100 units/ml penicillin and 100 μg/ml of streptomycin with 5 mM propionate and cultured at 37 °C for 24 h. Cells were washed with cold PBS (2 × 10 ml) in a fume hood and 1 ml triazole (Invitrogen, 15596026) was added. Cells were scraped and the suspension was transferred to a 1.5 ml Eppendorf tube and stored at −80 °C until further use.

RNA was isolated using the triazole-chloroform extraction method as described by triazole user guideline (11596026) by Invitrogen. RNA was reverse transcribed using an MMLV reverse transcriptase (Thermo Fisher) at 70 °C. Sequences of primers used for qPCR analysis with Radiant Green qPCR master mix (Applied Biosystems) are reported in Table S2. The data were analyzed using the 2^(-ΔΔCt)^ method to calculate relative mRNA levels for each gene of interest (41).

### Western blot analysis

Cells were grown in 6-well or 10-cm plates to a confluency of ∼50%. For propionate treatment, culture media was exchanged with EBSS media containing 5% FBS and 100 units/ml penicillin and 100 μg/ml of streptomycin with 5 mM propionate and grown in the humidified atmospheric chamber at 37 °C with 5% CO_2_ for 24 h. Cells were washed with PBS and detached by treatment with 0.05% trypsin-EDTA (w/v), for 10 min at 37 °C. The sample was resuspended in EBSS media, centrifuged at 1600 × *g* and the cell pellet was resuspended in 200 μl lysis buffer (20 mM HEPES, pH 7.5, 25 mM KCl, 0.5% Nonidet P-40 (^v^/_v_), 1% (^v^/_v_) protease inhibitor for mammalian tissues (Sigma)) per 100 mg pellet wet weight. Cells were lysed with three freeze-thaw cycles and the suspension was centrifuged at 12,000*g* to collect the supernatant. The solubilized proteins were separated on a 10 to 12% SDS-polyacrylamide gel and transferred to a nitrocellulose membrane, blocked with 5% milk in tris-buffer saline with 0.1% tween for ∼1 h at room temperature on a rocker. The protein levels of propionyl-CoA carboxylase (PCCA), short-chain acyl-CoA dehydrogenase (ACADSB), and (*R*)-methylmalonyl-CoA mutase (MMUT) was detected by Western blot analysis. Rabbit polyclonal PCCA (Abcam, ab187686), polyclonal SBCAD (Abcam, ab99951), and monoclonal MMUT (Abcam, ab134956) antibodies were used at a dilution of 1:1000. A secondary rabbit antibody conjugated to horse radish peroxidase (Kindlebio, #R1006) was used at 1:1000 dilution with the peroxide:luminol (1:1) as chemiluminescence substrate (Kindlebio, #R1002). Purified recombinant human MMUT was used as a positive control for MMUT.

### Sample preparation for metabolomic and lipidomic analysis

HT-29 cells were grown to a ∼40% confluency in 6-well plates and then exchanged with EBSS media containing 5% FBS and 0.2 mM propionate (lipidomics) or 5 mM propionate (metabolomics), 100 units/ml penicillin and 100 μg/ml streptomycin. Cells (∼10^6^ cells in 2 ml EBSS medium/well) were grown for 48 h at 37 °C in a humidified chamber containing 5% CO_2_. To collect cells, the culture medium was removed, and cells were washed with 2 × 2 ml PBS and treated with 400 μl of 0.05% trypsin-EDTA (∼10 min at 37 °C). Once the cells were detached, they were resuspended in 1 ml of culture medium and transferred into a centrifuge tube. Cells were washed with 1 ml PBS and centrifuged at 1600 × *g* for min to collect any remaining cells on the plate. Samples were washed with 1 ml PBS in the Eppendorf tube and centrifugation was repeated. The cells pellets were frozen on dry ice, and stored at −80 °C.

### Metabolomics data acquisition and analysis

Metabolite extraction and analysis were performed commercially, using the TM-widely targeted metabolomics services of Metware Biotechnology Inc. An Ultra High-Performance Liquid Chromatograph column (Waters ACQUITY UPLC HSS T3 C18, 1.8 μm, 2.1 mm × 100 mm) and triple quadrupole linear ion trap (QTRAP) mass spectrometer equipped with an ESI Turbo Ion Spray interface was used to acquire MS/MS spectra at the Metware facility. Unsupervised principal component and Pearson correlation coefficient analyses were performed within the R package. Differential changes in metabolites were determined by *p*-value (<0.05, Student’s *t* test).

### Lipidomics analysis

Total lipid extracts from cell pellets were prepared using a modified MTBE lipid extraction protocol (42). Briefly, 400 μl of cold methanol and 10 μl of internal standard mixture (mix EquiSPLASH, five fatty acid mixture and four ceramides mixtures; the final concentration of the internal standard mixture was 33.3 μg/ml for each lipid) were added to each sample. The sample was incubated at 4 °C, 650 rpm shaking for 15 min. Next, 500 μl of cold MTBE was added followed by incubation at 4 °C for 1 h with 650 rpm shaking. Cold water (500 μl) was added slowly, and the resulting extract was maintained 4 °C, 650 rpm shaking for 15 min. Phase separation was completed by centrifugation at 8000*g* for 8 min at 4 °C. The upper, organic phase was removed and set aside on ice. The bottom, aqueous phase was re-extracted with 200 μl of MTBE followed by 15 min incubation at 4 °C with 650 rpm shaking. Phase separation was completed by centrifugation at 8000*g* for 8 min at 4 °C. The upper, organic phase was removed and combined with a previous organic extract. The latter was dried under a steady stream of nitrogen at 30 °C. The recovered lipids were reconstituted in 100 μl of acetonitrile:isopropanol:water (1:2:1, v/v/v).

Total lipid extracts were analyzed by liquid chromatography coupled to targeted tandem mass spectrometry (LC-MS/MS). The LC-MS/MS analyses were performed on an Ultimate 3000 Ultra High-Performance Liquid Chromatograph coupled to a Thermo TSQ Altis Tandem Quadrupole Mass Spectrometer (Thermo Scientific, San Jose, CA). LC-MS/MS methodology was adapted from the literature (43). The separation was achieved using an ACQUITY Amide BEH column (1.7 μm; 2.1 × 100 mm) column (Waters, Milford, MA) maintained at 45 °C. Mobile phase compositions for solvents A and B consisted of ACN/H_2_O (95:5, v/v) and (50:50, v/v) respectively, with 10 mM ammonium acetate. The gradient profile had a flow rate of 0.6 ml min^−1^ and ramped from 0.1 to 20% B in 2 min, from 20 to 80% B in 3 min, dropped from 80 to 0.1% B in 0.1 min, and held 0.1% B for 2.9 min. Total chromatographic run time was 8.0 min. The injection volume was 2 μl. The auto-sampler was maintained at 7 °C. Electrospray ionization was achieved using either negative or positive mode. Mass spectrometry detection was done using selective reaction monitoring where predetermined precursor to product ion transitions were used. ESI source parameters were set as follows: voltage 3500 V in positive mode and −2500 V in negative mode, sheath gas (Arb) = 60, aux gas (Arb) = 15, sweep gas (Arb) = 1 and ion transfer tube temperature of 380 °C. Nitrogen was used as the nebulizer and argon as collision gas (1.5 mTor). The vaporizer temperature was set to 350 °C. Collision energies and RF lens voltage were optimized for each lipid class reference standard and supported by literature (43). LC-MS/MS data was acquired using Thermo’s Xcalibur software and data processing was achieved using Xcalibur 4.2 and TraceFinder 5.1. Additional data analysis was done using Prism 6 (GraphPad) and MetaboAnalyst (44).

We note that the targeted LC-MS/MS method for lipid assignments involved using predetermined chromatographic retention times and diagnostic precursor-to-product ion transitions (SRM, MS/MS). The HILIC LC is based on head group hydrophilicity, which eliminates the isomeric and isobaric overlap between lipid classes, *e.g.*, phosphoethanolamine (PE) is chromatographically separated from phosphocholine (PC), greatly reducing misidentification. The lipid IDs was confirmed by specific SRM transitions that provide structural information about the precursor and product ion. For example, the identification of the odd-length fatty acyl PE (PE 17:0_22:5, Fig. 6*C*) had a targeted retention time of 2.01 min and a negative ion SRM transition of *m/z* 778.5 to 269.2. The *m/z* 778.5 value corresponded to the deprotonated ([M-H]^-^) precursor ion for PE(39:5) (sum composition). The targeted (pre-determined) retention time allowed us to assign this precursor to the PE lipid class. The *m/z* 269.2 product ion corresponded to a deprotonated FA17:0 which allowed us to provide acyl chain information to the sum composition. If one of the acyl chains is 17:0, then the other has to be 22:5. Note, that while there are other possible acyl chain configurations for PE(39:5), these lipid structures would have a different product ion for the SRM. Another important point in lipid identification was the choice of the ionization mode used for detection. Information on glycerophospholipid fatty chains was obtained by detecting them in the negative ion mode, which yields acyl chain-specific product ions by MS/MS. Therefore, each lipid assignment was been confirmed by chromatographic retention time and MS/MS analysis, which provides high confidence in our structure determination for all reported lipids, including those containing odd-chain fatty acyls.

## Data availability

All data with the exception of the lipidomc data, are contained within the manuscript. The lipidomic data has been made available *via* Mendeley data repository under the following reference: Jones, Jace (2024), “Targeted lipidomics on HT-29 cells that were treated with and without propionate”, Mendeley Data, V1, https://doi.org/10.17632/7vpbms74cb.1.

## Supporting information

This article contains supporting information.

## Conflict of interest

The authors declare that they have no conflicts of interest with the contents of this article.
