# Supplementary material for: Differential utilization of vitamin B12-dependent and independent pathways for propionate metabolism across human cells
Source: J Biol Chem. 2024 Aug 14;300(9):107662. doi: 10.1016/j.jbc.2024.107662 (PMC11408853; doi:10.1016/j.jbc.2024.107662)
Supplement: Supporting information [file mmc1.docx]

**Supporting Information**

**Differential utilization of vitamin B_12_-dependent and independent pathways for propionate metabolism across human cells**

Harsha Gouda^1^, Yuanyuan Ji^2^, Sneha Rath^3,4^, David Watkins^5^, David Rosenblatt^5^, Vamsi Mootha^3,4^, Jace W. Jones^2^, and Ruma Banerjee^1^*

^1^Departments of Biological Chemistry, University of Michigan, Ann Arbor, MI 48109, ^2^Department of Pharmaceutical Sciences, University of Maryland School of Pharmacy, Baltimore, MD 21201, ^3^Howard Hughes Medical Institute and Department of Molecular Biology, Massachusetts General Hospital, Boston MA 02114, ^4^Broad Institute, Cambridge, MA 02142,^5^Department of Human Genetics, McGill University, Montreal, QC H3A 1B1, Canada,

*Corresponding author. Email:rbanerje@umich.edu

**Table of Contents**

Figure S1. Validation of MMUT CRISPR KO.

Figure S2. Western blot analysis of MMUT, ACADSB and PCCA

Table S1. Metabolomics data for propionate-induced changes in HT-29 cells

Table S2. Primers used for relative quantification of mRNA levels of gene of interest using quantitative PCR analysis

**
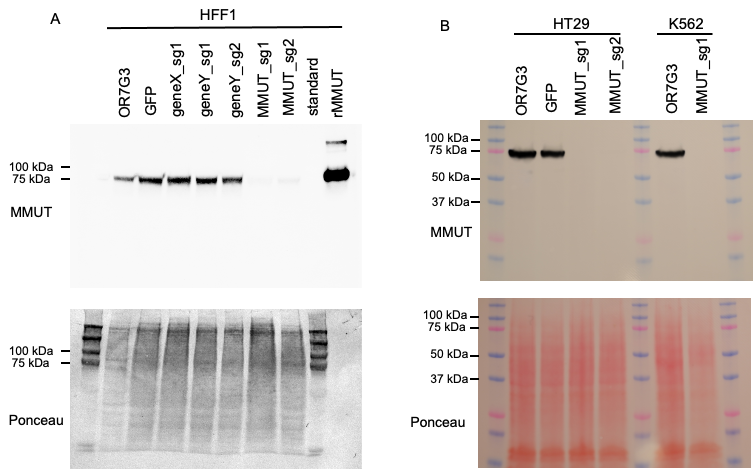
**

**Figure S1. Validation of MMUT CRISPR KO.** A-B, Validation of MMUT CRISPR KO in HFF1 (A), HT29 and K562 (B) cells. OR7G3 and GFP guide sequences were used as controls. The lower panels show Ponceau staining for equal loading. MMUT sg1 and sg2 denote guide sequences used for MMUT KO, rMMUT denotes purified recombinant human MMUT.

**
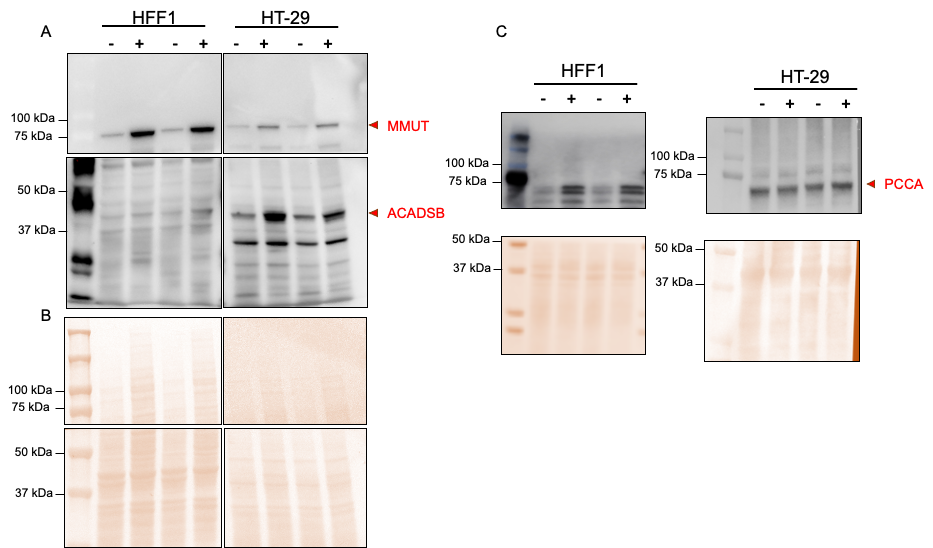
**

**Figure S2. Western blot analysis of MMUT, ACADSB and PCCA.** A, The top half of the membrane was probed with rabbit monoclonal MMUT (Abcam, ab134956) while the bottom half was probed with the rabbit polyclonal ACADSB antibody (Abcam, ab99951) in samples from the indicated cell lines, which were either untreated (-) or treated (+) with 5 mM propionate for 24 h. B, Ponceau staining of the nitrocellulose membrane in A to assess equal loading. C, Representative nitrocellulose membranes probed with rabbit polyclonal PCCA antibody (Abcam, ab187686) in lysates derived from HFF1 (A) and HT-29 (B) cells treated with propionate as described in A. These figures are the full images of the blots shown in Fig. 4C in the main text.

| Gene of interest | Forward primer | Reverse primer |
| --- | --- | --- |
| ACTIN | 5'-TCCCTGGAGAAGAGCTACGA-3' | 5'-AGCACTGTGTTGGCGTACAG-3' |
| GAPDH | 5'-AATCCCATCACCATCTTCCA-3' | 5'-TGGACTCCACGACGTACTCA-3' |
| TBP | 5'-TGTATCCACAGTGAATCTTGGTTG-3' | 5'-GGTTCGTGGCTCTCTTATCCTC-3' |
| ACADSB | 5'-TATGCATCAGAGATTGCAGG-3' | 5'-AGGGTAATCTTTGGTGTAGC-3' |
| ECHS1 | 5'-GAAGACCTTCGAGGAGGAC-3' | 5'-GATATCAGCTCCAGCTGCA-3' |
| HIBCH | 5'-ATTACAATGGGTGGGGGAGT-3' | 5'-CCAAGTTTTCCTTGGAGTCG-3' |
| ADHFE1 | 5'-GAGTCATACACCACCCTGCC-3' | 5'-CTGACAGCCCTCTTCAGATAC-3' |
| ALDH1A6 | 5'-AATGTTCTCATTCACCGGC-3' | 5'-GTGTAGAATTGGATGCCCTG-3' |
| PCCA | 5'-ACAACAATGGCGGGGTTCT-3' | 5'-ACGGGACACCATTAAGCACT-3' |
| MMUT | 5'-AGTCCAAAACAGGCCTACCAG-3' | 5'-CGTTGCTGTATGAGCCTGGA-3' |
| CS | 5'-CCTGATGAGGGCATCCGTTT-3' | 5'-GTTCTTCCCCACCCTTAGCC-3' |

**Table S2**: **Primers used for relative quantification of mRNA levels of gene of interest using quantitative PCR analysis.**
